# Current Perspectives on In Vivo Noninvasive Tracking of Extracellular Vesicles with Molecular Imaging

**DOI:** 10.1155/2017/9158319

**Published:** 2017-01-29

**Authors:** Prakash Gangadaran, Chae Moon Hong, Byeong-Cheol Ahn

**Affiliations:** Department of Nuclear Medicine, Kyungpook National University School of Medicine and Hospital, Daegu 700-721, Republic of Korea

## Abstract

Clinical and preclinical in vivo tracking of extracellular vesicles (EVs) are a crucial tool for the development and optimization of EV-based diagnosis and treatment. EVs have gained interest due to their unique properties that make them excellent candidates for biological applications. Noninvasive in vivo EV tracking has allowed marked progress towards elucidating the mechanisms and functions of EVs in real time in preclinical and clinical studies. In this review, we summarize several molecular imaging methods that deal with EVs derived from different cells, which have allowed investigations of EV biodistribution, as well as their tracking, delivery, and tumor targeting, to determine their physiological functions and to exploit imaging-derived information for EV-based theranostics.

## 1. Introduction

The naturally produced biological nanoparticles, termed extracellular vesicles (EVs), are released from most of cells into the extracellular space. These include exosomes (40–100-nm diameter membranous vesicles of endocytic origin) and microvesicles (large membranous vesicles of 50–500-nm diameter), which are shed directly from the plasma membrane [1, 2]. Proteins and lipids are the main components of EV membranes, which are enriched with lipid rafts [3]. EVs are capable of carrying various biological materials such as lipids, proteins, mRNA, and miRNA [3–6]. A previous study has also demonstrated that pancreatic cancer cell-derived EVs can contain fragments of double-stranded genomic DNA [7]. Intercellular communication is essential to cell development and maintenance of homeostasis in multicellular organisms. These communications between cells can be localized or distant. Distant intercellular communication in particular is achieved via EVs [8, 9]. A major discovery has been that the cargo of EVs included both mRNA and miRNA; mRNAs could be translated into proteins in target cells [6, 10].

The recent studies showed that the biological roles of EVs ranged from normal physiological functions, such as stem cells in kidney monitoring and repair [11, 12], immune modulation [13], and tissue homeostasis [14], to contributing to the pathophysiology of several diseases [10]. EVs can modulate immune-functional properties against tumors and set up tumor escape mechanisms [15]. EVs are ideal vehicles for molecule-delivery (suicidal proteins and RNAs, small molecule drugs, etc.) to certain cells, because of their biocompatibility, stability in blood circulation, and, most importantly, their ability to target certain cell types [16]. Even though the first discovery of EV's role in cell-cell communication was made in the 1990s [17], more discoveries continue to be made. During the past few years, there has been enormous progress in our understanding of the function of EVs and their possible applications in clinical settings [18], yet more biological roles remain to be discovered. The role of EVs in in vivo communication and their applicability as vehicles for drug-delivery to targets require the investigation of their biodistribution.

Noninvasive imaging modalities have the potential of providing better understanding of the biological process and effectiveness of EVs for various diseases by determining the in vivo kinetics of EVs. Molecular imaging are mainly categorized into two main technique classes, namely, direct and indirect labeling. Direct labeling involves labeling of EVs by means of various agents, such as magnetic particles [19], lipophilic tracer dyes [20], or radionuclides [21], while, in indirect labeling, cells are genetically altered to transcribe and translate reporter proteins, and the EVs containing the reporter proteins are then isolated [22, 23].

Here, we will review studies that used molecular imaging techniques to evaluate EV visualization, biodistribution, and targeted drug-delivery in certain diseases and further discuss important issues in this area. We also provide a general overview and specific examples of in vivo tracking of EVs by means of various imaging modalities, to enhance understanding of the roles of EVs in various pathophysiological conditions.

## 2. Biogenesis of Extracellular Vesicles

EVs are secreted from various cells, such as immune cells (T and B cells, dendritic cells, natural killer cells, monocytes/macrophages, platelets, and red blood cells [RBCs]) [24–29]; mesenchymal stem cells (MSCs) [14]; and tumor cells (glioblastoma, thyroid, lung, breast, liver, ovarian, and colon cancers) [30–35]. Bacteria and plants also secrete EVs [36, 37]. Even though almost all cells are able to release EVs with various biological and pathological functions, they were considered as “garbage bags” in the past.

### 2.1. Exosomes

**Figure 1:**
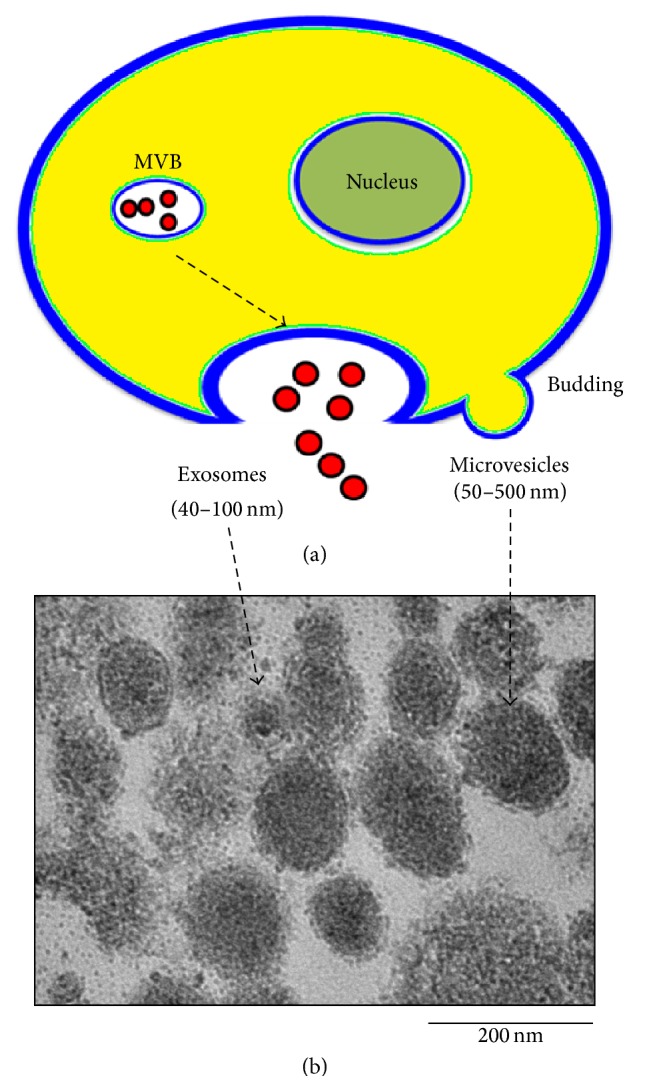
Release of exosomes and microvesicles. (a) Exosomes are represented by small vesicles of different size released from multivesicular body and microvesicles bud directly from the plasma membrane. (b) Typical structure of EV by TEM images and the size of EVs is around 40–500 nm. EV; extracellular vesicle, TEM; transmission electron microscopy.

Endocytic membrane trafficking is controlled through several cytosolic regulatory mechanisms that dictate the number, composition, and fate of vesicles in their lumen (i.e., intraluminal vesicles, ILVs) and exosomes [44, 45]. Multivesicular bodies (MVBs) (Figure 1(a)), which appear along the endocytic pathway, are characterized by the presence of ILVs formed by inward budding of the outer cell membrane [46]. Exosome biogenesis and cargo sorting involves the coordinated recruitment and employment of endosomal sorting complex required for transport (ESCRT) machinery and its associated proteins [47–49]. A study by Ostrowski et al. established that a number of Rab family proteins (including Rab27a and Rab27b) act as vital regulators of exosome release [50]. However, the mechanism behind this process is not yet completely understood.

### 2.2. Microvesicles

The small vesicles are shed from the surface of many cells called microvesicles (Figure 1(a)) [51]. Microvesicles are slightly larger than exosomes and can therefore carry more cargo load than exosomes. EVs (exosomes and microvesicles) can be identified under electron microscopy by their round shape and the presence of a lipid bilayer (Figure 1(b)).

## 3. Emerging Therapeutic and Diagnostic Potential of Extracellular Vesicles

Recent studies have revealed the biopathological roles of EVs in treatment, drug-delivery, tumor progression, and disease diagnosis [52]. Recently, the therapeutic application of EVs has also emerged [53]; for example, the application of dendritic cell- (DC-) derived exosomes for cancer treatment was recently investigated in a clinical trial (NCT01159288) [54, 55]. MSC-EVs have been proposed as a replacement for MSCs for the treatment of various diseases, such as for tumor inhibition and cardiac and brain injuries [56–60]. MSC-derived exosome for Crohn's fistula treatment was recently under investigation in a clinical trial [61, 62].

The emerging evidence that EVs possess special characteristics may indicate that they can be used to create an EV-based drug-delivery system that is superior to synthetic drug carriers [53]. A previous study used exosomes for delivering curcumin as a treatment for an inflammatory disease [63]. To enhance the function of drugs in the central nervous system, exosomes can also carry small molecular drugs across the blood-brain barrier [64].

Tumor-derived EVs may enhance tumor cell activity. For example, exosomes released from breast carcinomas stimulate cancer cell movement, leading to establishment of distant metastasis [65]. Recent studies showed that tumor-derived EVs promote endothelial cell migration during angiogenesis in the tumor microenvironment via ERK1/2 and JNK signaling pathways [66]. Numerous studies have shown that tumor-derived EVs transfer oncogenic activity, thus promoting tumor progression [67, 68]. Tumor-secreted exosomes have been shown to facilitate tumor progression by affecting the adhesion of the primary cancer cells and promoting metastasis [65, 69].

Recently, other biomarker-like exosomes have been investigated. Glypican-1 and endothelial locus-1 positive exosomes may serve as potential noninvasive diagnostic tools for detecting the early stages of pancreatic cancer and breast cancer, respectively [70, 71]. Multiple research groups are actively seeking novel biomarkers, including EV-based markers, for detecting hidden cancers at the earliest possible stage.

## 4. Molecular Imaging Techniques for Tracking Extracellular Vesicles

**Figure 2:**
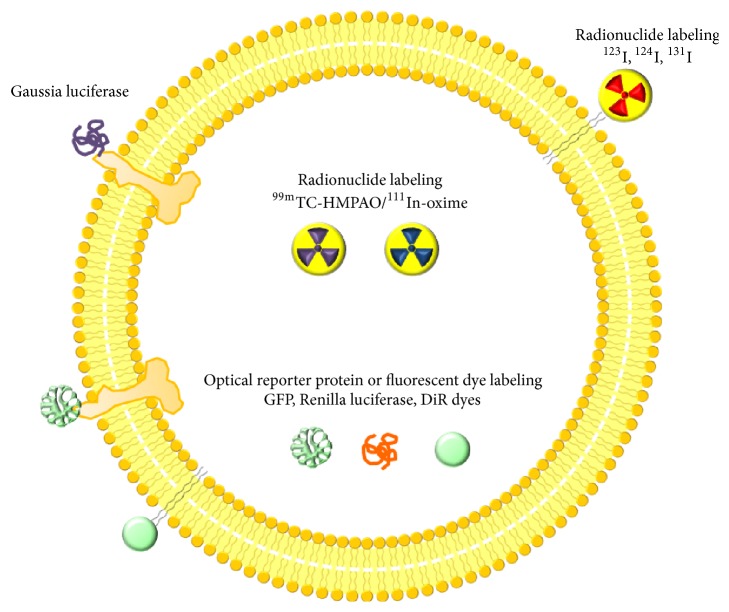
Strategy for labeling of extracellular vesicles. GFP; green fluorescent protein, DiR; near infrared fluorescent dye.

Methods that allow EV monitoring in vivo offer several advantages over the traditional ex vivo methods, which require sacrifice of the animal and histological analysis. Molecular imaging, for example, is fundamentally noninvasive and allows for quantitative assessment of the EV biodistribution and the effects of EV therapy over time (Figure 2). Bioluminescent imaging (BLI) and fluorescence imaging (FLI) have the advantage of high-throughput efficiency at low cost.

BLI uses light generated from a luciferase enzyme-substrate and an ultrasensitive cooled charge-coupled camera for signal detection. In indirect labeling of EVs, the cells are first transfected with a vector containing imaging reporter genes (e.g., Gaussia luciferase [Gluc]). These cells then produce* Gluc* mRNA which is translated into the reporter protein. Isolation of EVs from these cells will provide EVs containing the reporter protein from the cell. In stably transfected cells, the reporter gene is inherited by daughter cells upon cell division. This strategy is essential for long-term isolation of EVs containing the reporter protein.

FLI signal generation is achieved by exciting the fluorescent proteins/dye at a given light wavelength and detection of the light emission at another wavelength by means of a charge-coupled camera. In indirect labeling of EVs with a fluorescent protein (GFP/RFP) [23, 72], the same method is used as that for bioluminescent reporter proteins. Direct labeling of EVs with a fluorescent dye is a very simple technique to perform. EVs can be washed a few times after incubation with a fluorescent lipophilic dye [12, 73, 74], and they are then ready for in vivo experiments. Direct labeling of EVs with fluorescent dyes has a few drawbacks. The exogenous dye labeling produces a nonspecific signal due to the long half-life of the fluorescent dye and its resistance to degradation. One of the main considerations when a fluorescent dye is used to label the membranes of the EVs is that the dye can be released from the EVs; this can lead to generation of non-EV-associated signals [74–76]. Compared to BLI, FLI is generally less sensitive, due to the higher background signal. In most cases, FLI is used for live imaging of shallow tissue areas.

Nuclear imaging is also a widely used molecular imaging technique. As radionuclides emit gamma rays or positrons, radionuclides can be detected, even when located deep in organs. Radionuclides can be placed inside EVs [20, 21] or be incorporated as a label in membrane proteins of EVs [41, 42].

Magnetic resonance imaging (MRI) can be used for tracking EVs after labeling with ultrasmall superparamagnetic iron oxide nanoparticles (USPIO, 4–6 nm) [20, 43]. As MRI provides high-resolution anatomical images, we can easily discriminate the location of the EVs. However, it is limited by its relatively lower sensitivity of detection than that of other molecular imaging modalities [77].

## 5. Tracking Extracellular Vesicles by Bioluminescence Imaging

BLI is a powerful method for cell tracking in small animals (such as mice) over time, without requiring the subject to be euthanized [78–80]. Bioluminescence reporters are able to reveal the in vivo biodistribution and allow tracking of EVs with very high sensitivity. To date, there have been very few studies that have used bioluminescence reporters for in vivo visualization and tracking of the distribution of EVs (Table 1) [22, 23, 38].

### 5.1. Direct Labeling Methods

To date, there has been no report about direct bioluminescence reporter-based labeling of EVs. Yet, it is possible to achieve labeling of EVs with bioluminescence reporters. A few other studies have shown that exogenous peptide, proteins, and siRNA can be loaded into EVs [81–83]; similarly, it is possible to load exogenous bioluminescence reporter proteins into EVs, which could be used for visualizing and monitoring EVs in vivo in the near future.

### 5.2. Indirect Labeling Methods

Gluc is a reporter protein that emits bioluminescence when its substrate, coelenterazine, is present, and lactadherin is a membrane-associated protein mainly found in exosomes. This is referred to as Gluc-lactadherin which is eventually found in the exosomes [23]. A previous study used melanoma cell (B16-BL6) to produce exosomes, in which they transfected cells with a Gluc-lactadherin construct. Intravenous injection of these exosomes revealed for the first time that EVs could be visualized in vivo by using bioluminescent reporter proteins present in exosomes. This study provided the overall tissue distribution and quantitative pharmacokinetic properties of exosomes in vivo and proved that exosomes have very short half-lives after systemic administration. This was not possible with dye-based EV studies, as dye can be released from the EVs, which can lead to non-EV-associated signals [23].

In another study, Gluc was fused to a biotin acceptor domain in cells, which then produced the labeled EVs. Intravenous injection of EVs derived from these cells revealed that the EVs could be visualized in vivo and showed an organ-specific distribution of EVs. The Gluc signal was observed in the spleen and liver in the EV-injected mice and later in the liver and kidney of these mice. This was eventually quantified by determining the average bioluminescent radiance in organ regions of the mice. The advantage of this study was that it combined both Gluc and biotin to create an EV-specific reporter with a high sensitivity for studying in vivo dynamics of systemically administered EVs. The BLI revealed that most EV-Gluc was cleared from the animals by 6-h after injection and also showed that EVs could be targeted to tumors [22].

Another study also used Gluc-lactadherin for exosome labeling. It clearly indicated that macrophages play a major role in the clearance of exosomes in general, by using BLI [38]. All three studies showed rapid in vivo distribution of EVs in animals.

## 6. Tracking Extracellular Vesicles by Optical Fluorescence

The strategy for imaging and tracking for EVs by labeling them fluorescently has been widely used to investigate in vivo behavior of exogenous EVs both in vitro and in vivo (Table 1) [12, 20, 39, 40, 72, 84–86].

### 6.1. Direct Labeling Methods

**Figure 3:**
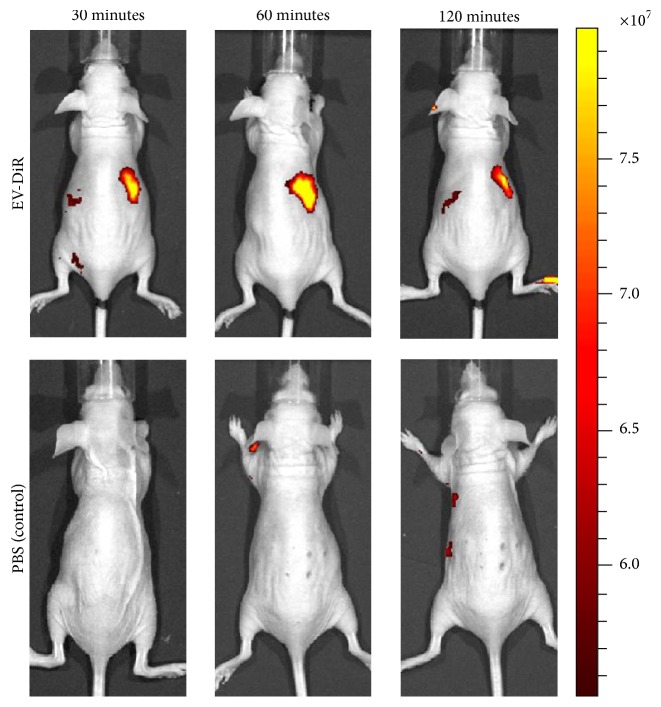
In vivo noninvasive visualization of fluorescent dye labeled EVs in nude mice. Representative in vivo fluorescent imaging of EV-DiR or PBS (control) was administered via the tail vein in nude mice. Images were acquired at 30, 60, and 120 min after injection. EV, extracellular vesicle.

The direct labeling protocol is very simple and there is no need to use genetically modified EVs (Figure 3). This simple imaging strategy, which uses dye to label the lipid membrane of EVs, has been used to reveal the spatiotemporal location of systemically injected exogenous EVs in organs and target tumors [12, 40]. Grange et al. have demonstrated that it is possible to analyze the biodistribution of EVs by direct labeling of EVs with a DiD dye. In particular, they observed that the labeled MSC-derived EVs were localized within injured kidneys in vivo. The signal generated by the EVs was maintained even 24 h after the injection [12].

Hood et al., using a lipophilic tracer dye (DiD), showed that the melanoma exosome prepares the way for metastasis of this cancer to the sentinel lymph nodes [84]. Another study used DiR labeling of EVs to show delivery of Let-7a miRNA by exosomes to epidermal growth factor receptor-expressing breast cancer cells in vivo, which was confirmed ex vivo [40]. Smyth et al. compared the biodistribution and delivery efficiency of tumor-derived exosomes and liposomes by using a lipophilic dye (DiR). FLI revealed that tumor-derived exosomes and liposomes had a similar clearance time in vivo. Furthermore, this study also revealed that liposomes and tumor-derived exosomes were not targeted to the tumor when injected systematically. Moreover, FLI showed that tumor-derived exosomes and liposomes injected into the tumor stayed within the tumor [20].

Wiklander et al. used a near-infrared dye (DiR) and studied the biodistribution profile of EVs derived from a broad range of different cell types, namely, HEK293T, primary mouse DCs, and primary human MSCs. DiR-labeled HEK293T EVs were subsequently injected via the tail vein of mice, but the fluorescent signal in whole mouse imaging did not yield sufficient accuracy to determine from which tissue or organ the signal originated; thus, organs were harvested and imaged ex vivo. They also showed that different routes of injection (intravenous, intraperitoneal, and intramuscular) yielded different distribution patterns for EV signals from organs and tissues. Furthermore, FLI revealed that distributions of EVs derived from different cells yielded different distribution patterns in internal organs. They also assessed how targeted EVs as well as tumor burden influenced the biodistribution [75]. Nevertheless, the use of fluorescent proteins (GFP/RFP) limits the visualization of EVs in vivo. Therefore, this technique cannot be applied to visualize EVs.

A few other studies have used lipophilic dyes to label EVs in order to study their in vivo properties [12, 40, 75, 87, 88]. However, these dyes, including DiR, DiD, PKH26, and PKH67, are reported to have an in vivo half-life ranging from 5 to >100 days. Where the administered EVs have been visualized in vivo, the persistence of the dye may outlast the labeled EVs in vivo. In addition, labeling of EVs with exogenous signaling agents can result in changes to the homing characteristics of EVs, due to the labeling procedures used.

### 6.2. Indirect Labeling Methods

In indirect labeling, the transgene used encodes a fluorescent protein, which acts as an intrinsically produced reporter protein. Similar approaches for visualizing EVs have been proposed, and a few studies have exploited imaging based on fluorescent proteins, such as GFP, RFP, and dTomato, to study EVs both in vitro and in vivo [23, 72, 85, 86]. A few studies have used biomarkers in EVs, such as CD63, which was used to design a reporter conjugated to fluorescent proteins (GFP/RFP) [86, 89]. The efficiency of in vivo imaging of EVs with a fluorescent signal in mice depends on the gene expression level in the cells [90]. Unfortunately FLI of intravenously administered EVs is problematic, due to the low signal yield of the fluorescence-labeled EVs [91].

## 7. Tracking Extracellular Vesicles by Nuclear Imaging

Nuclear imaging is widely used for preclinical and clinical cell trafficking [77]; some nuclear imaging studies for in vivo EV monitoring (Table 1) have been published [20, 21, 41, 42]. The knowledge that has been accumulated for tracking cells with nuclear techniques can also be applied to tracking EVs. Although FLI and BLI cannot be used for effective visualization of EVs located in deep organs or tissues, due to limitation of tissue penetration of optical fluorescent and bioluminescent signals, nuclear imaging are able to visualize EVs, regardless of their location in the body, due to the excellent tissue penetration characteristics of gamma rays [91].

### 7.1. Direct Labeling Methods

**Figure 4:**
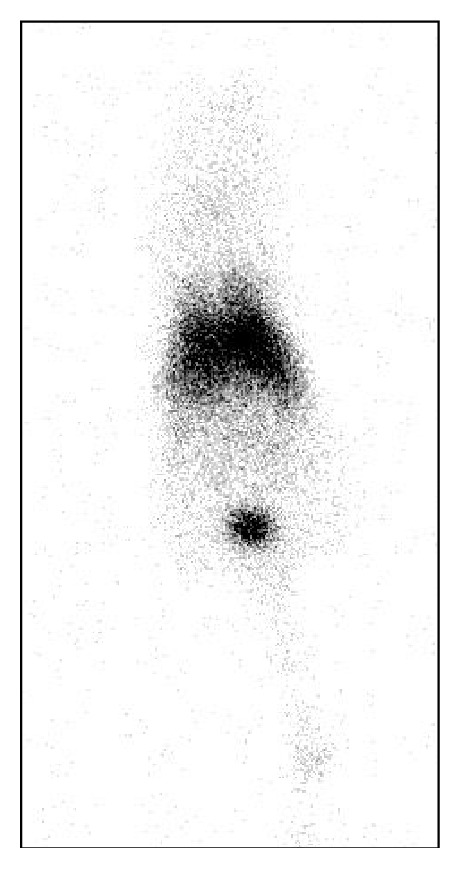
In vivo noninvasive visualization of ^99m^Tc-HMPAO labeled extracellular vesicles (EVs) in nude mice. ^99m^Tc-HMPAO labeled EVs were administrated via tail vein. Image was acquired using pin-hole gamma camera at 1 hour after injection. The image shows liver and spleen uptake. But there was no tracer uptake in the thyroid gland and stomach, which were visualized in free ^99m^Tc images.

^111^In-oxine and ^99m^Tc-hexamethylpropyleneamineoxime (HMPAO) were widely used for labeling white blood cells in preclinical and clinical studies, and these were also applied for tracking EVs [20, 21]. Smyth et al. labeled EVs using ^111^In-oxine and fluorescent dye and showed similar distribution patterns [20]. ^111^In-oxine could be visualized using a gamma camera or by single-photon emission computed tomography (SPECT); ^111^In-oxine was used for determining biodistribution only. As free ^111^In-oxine accumulates in the reticuloendothelial system, it is hard to distinguish whether it has been released from EVs [91]. Hwang et al. successfully labeled EVs using ^99m^Tc-HMPAO [21]. ^99m^Tc-HMPAO-labeled EVs are easily visualized using a gamma camera or SPECT. As free ^99m^Tc-HMPAO shows a high brain uptake and free ^99m^Tc is taken up by the thyroid and salivary glands, the freed form can be easily discriminated from the labeled form (Figure 4). However, a drawback of this method is the low radiochemical yield at low EV concentrations [20, 91].

Recently, ^99m^Tc-tricarbonyl was used for labeling EVs. ^99m^Tc-tricarbonyl has also been used for the labeling of a wide range of biomolecules, and it binds to several amino acids, such as histidine, methionine, and cysteine [41]. ^99m^Tc-tricarbonyl-labeled EVs can be visualized using a gamma camera or SPECT. This method showed relatively higher labeling efficiency in RBC-derived EVs (38.8%). However, that study only obtained 1-h images and performed image-based analysis; therefore, further investigations may be needed to track EVs over a longer time when using this method.

Radioiodine (^123^I, ^124^I, ^125^I, and ^131^I) is commonly used for diagnosis and treatment of thyroid disease [92] and for tracking cells [93, 94]. Gamma camera and SPECT images can be obtained using ^123^I or ^131^I and positron-emission tomography (PET) images using ^124^I. Iodination of surface protein of the cells or transfection of sodium iodide symporter (NIS) to the cells was used for tracking the cells. Similar methods could be applied to monitor EVs in vivo. To establish more stable radioiodine-labeled EVs, a streptavidin-lactadherin fusion protein expressed in EVs was conjugated with a ^125^I-labeled biotin derivative [42]. Gamma camera or PET images can be obtained by changing ^125^I-labeled biotin to ^123^I-labeled biotin or ^124^I-labeled biotin.

### 7.2. Indirect Labeling Methods

Unfortunately, there are no articles about indirect radionuclide labeling EVs. Indirect radionuclide labeling methods might provide additional information. For indirect labeling, reporter gene transductions to cells before producing EVs are needed. Many kinds of nuclear reporters are already established and selection of appropriate reporter is crucial for successful in vivo EV monitoring using nuclear imaging.

## 8. Tracking Extracellular Vesicles by Magnetic Resonance Imaging

MRI is widely used in clinics for visualizing anatomical structures with high-resolution images. Recently, EVs were labeled with USPIOs, which shows decreased signal intensity in T2-weighted images [19, 43]. Hu et al. reported that EVs were loaded with USPIOs using electroporation (54.9 *μ*g iron per 100 *μ*g EV protein) [43]. After injection of the EVs into the feet of the mice, MRI successfully visualized migration of the EVs to the draining lymph nodes (Table 1).

Busato et al. labeled parent cells (adipose stem cells) with USPIOs and collected EVs from these cells [19]. As they did not manipulate the membrane of the EVs, the integrity of the EV membranes was preserved. However, the iron contents of these EVs were lower than those reported in a previous study (0.643 *μ*g of iron per 100 *μ*g of EV proteins) [19, 43]. They only showed decreased signal intensity after intramuscular injection of the EVs. For tracking EVs, a large amount of EVs is needed to allow visualization on MRI. Although MRI shows high-resolution images, the sensitivity of USPIOs is relatively lower than that of optical imaging or nuclear imaging [77]. Additionally, it is difficult to discriminate EVs on MR images due to the decreased signal intensity achieved with the accumulation of USPIOs.

## 9. Translational and Clinical Applications

Recently, EV-mediated therapies have emerged, and several clinical trials are under investigation [53, 95]. Most of the clinical trials focus on visualizing the treatment effect of EVs. Visualizing the kinetics of EVs in the human body and quantifying the EVs delivered to target lesions could reduce unnecessary effort and expenses in trials. As indirect labeling methods require gene transduction into cells before gathering EVs, which could change the biological characteristics of the cells and might cause ethical issues for clinical applications, the method might not be appropriate for clinical trials. Optical imaging technologies suffer from the intrinsic limitations of signal penetration and therefore might not be effective in human applications. Nuclear imaging using direct labeling could be a more useful option for clinical applications, as it is safe and has no depth limitation. Radioiodine, ^99m^Tc, and other radionuclides are already widely used in clinics; and ethical and legal problems in a clinical translation study using in vivo monitoring of EVs can be avoided by using this method. In addition, nuclear imaging technology of EVs has the benefit of theranostic potential by changing gamma ray emitting diagnostic radionuclides (^123^I, ^124^I, ^99m^Tc, ^64^Ga, etc.) to beta-particle emitting therapeutic radionuclides (^131^I, ^90^Y, ^177^Lu, etc.) [94].

## 10. Conclusion

In vivo imaging of EVs is important for realizing the biology and pathophysiology of EVs and for application of EVs as part of the diagnostic and therapeutic approach in various diseases. Here, we described both direct and indirect EV-labeling strategies for bioluminescent, fluorescent, nuclear, and MR imaging. Gluc combined with transmembrane domains, such as lactadherin, could reveal the spatiotemporal distribution of EVs sensitively, in a quantitative manner, in small animals without background signals. Florescent dye labeling of EVs is easy to perform, but it has the inherent disadvantage of signal generation even after EV degradation. Optical imaging has a high sensitivity without high cost but is applicable only to small animals due to the depth limitation. Nuclear imaging modalities, such as PET, SPECT, and gamma camera imaging, have problems with labeling of radionuclides but provide excellent sensitivity and easier quantification and their clinical applications are feasible. Multimodal imaging, which combines the strengths of different imaging techniques to provide corresponding information on different features of the biological process under investigation, can offer a better solution to the technical disadvantages of individual imaging modalities. An appropriate and specific labeling strategy for use with EVs should be selected for each experimental setting.

## Figures and Tables

**Table 1:** Strategies of in vivo tracking of extracellular vesicles.

Imaging modality	Labeling strategy	Types of cells	Isolation method	Labeling method	Injection Site	Subject	Duration of tracking	Purpose	Clinical translation	Reference
BLI	Indirect	HEK 293T cells	UC	Gluc	IV	Mice	30 to 360 minutes	Biodistribution/Tumor Targeting	x	[22]
		Melanoma cell line	UC	Gluc	IV	Mice	10 to 240 minutes	Biodistribution	x	[23]
		Melanoma cell line	UC	Gluc	IV	Mice	10 to 240 minutes	Biodistribution	x	[38]

FLI	Direct	MSC	UC	DiD	IV	Mice	10 minutes to 24 hours	Targeting to injured kidney	x	[12]
		Mouse B16-F10 (CRL 6475) melanoma cells	UC	DiR	Intradermal	Mice	48 hours	Nodal trafficking	x	[39]
		HEK293	UC	DiR	IV	Mice	24 hours	Biodistribution/Tumor Targeting	x	[40]
		4T1, MCF-7, and PC3 cells	Sucrose density cushion/UC	DiR	IV and IT	Mice	30 minutes to 7 hours	Biodistribution/Tumor Targeting	x	[20]

NI	Direct	4T1, MCF-7, and PC3 cells	Sucrose density cushion/UC	^111^In-oxine	IV and IT	Mice	30 min to 7 hours	Biodistribution/Tumor Targeting	o	[20]
		Raw 264.7, HB1.F3	Nanovesicles (sequential filtration) iodixanol gradient/UC	^99m^Tc-HMPAO	IV	Mice	30 minutes to 5 hours	Biodistribution	o	[23]
		Erythrocyte	UC	^99m^Tc-tricarbonyl	IV	Mice	1 hour	Biodistribution	o	[41]
		B16BL6 murine melanoma cell line	UC	^125^I-biotin derivatives	IV	Mice	1 minute to 4 hours	Biodistribution	x	[42]

MRI	Direct	Mouse B16-F10 (CRL 6475) melanoma cells	UC	USPIO	Intradermal	Mice	1 hour, 48 hours	Nodal trafficking	o	[43]
		Murine adipose stem cell (C57BL/6)	PureExo® Exosome Isolation Kit	USPIO	IM	Mice	1 hour	Retention at injection site	o	[19]

BLI, bioluminescence imaging; FLI, fluorescence Imaging; NI, nuclear imaging; MRI, magnetic resonance imaging; UC, ultracentrifuge; MSC, mesenchymal stem cell; USPIO, ultrasmall super paramagnetic iron oxide; IV, intravenous; IT, intratumor; I.M, intramuscular; DiD and DiR, near-infrared dyes; Gluc, Gaussia luciferase.

## References

[1] Keller S., Sanderson M. P., Stoeck A., Altevogt P. (2006). Exosomes: from biogenesis and secretion to biological function. *Immunology Letters*.

[2] Grange C., Tapparo M., Collino F. (2011). Microvesicles released from human renal cancer stem cells stimulate angiogenesis and formation of lung premetastatic niche. *Cancer Research*.

[3] Mathivanan S., Fahner C. J., Reid G. E., Simpson R. J. (2012). ExoCarta 2012: database of exosomal proteins, RNA and lipids. *Nucleic Acids Research*.

[4] Hong B. S., Cho J.-H., Kim H. (2009). Colorectal cancer cell-derived microvesicles are enriched in cell cycle-related mRNAs that promote proliferation of endothelial cells. *BMC Genomics*.

[5] Yamamoto H. (2014). Detection of DNA methylation of gastric juice-derived exosomes in gastric cancer. *Integrative Molecular Medicine*.

[6] Valadi H., Ekström K., Bossios A., Sjöstrand M., Lee J. J., Lötvall J. O. (2007). Exosome-mediated transfer of mRNAs and microRNAs is a novel mechanism of genetic exchange between cells. *Nature Cell Biology*.

[7] Kahlert C., Melo S. A., Protopopov A. (2014). Identification of double-stranded genomic DNA spanning all chromosomes with mutated KRAS and p53 DNA in the serum exosomes of patients with pancreatic cancer. *The Journal of Biological Chemistry*.

[8] Yáñez-Mó M., Siljander P. R.-M., Andreu Z. (2015). Biological properties of extracellular vesicles and their physiological functions. *Journal of Extracellular Vesicles*.

[9] Lai C. P., Kim E. Y., Badr C. E. (2015). Visualization and tracking of tumour extracellular vesicle delivery and RNA translation using multiplexed reporters. *Nature Communications*.

[10] Ratajczak J., Wysoczynski M., Hayek F., Janowska-Wieczorek A., Ratajczak M. Z. (2006). Membrane-derived microvesicles: important and underappreciated mediators of cell-to-cell communication. *Leukemia*.

[11] Camussi G., Deregibus M. C., Tetta C. (2010). Paracrine/endocrine mechanism of stem cells on kidney repair: role of microvesicle-mediated transfer of genetic information. *Current Opinion in Nephrology and Hypertension*.

[12] Grange C., Tapparo M., Bruno S. (2014). Biodistribution of mesenchymal stem cell-derived extracellular vesicles in a model of acute kidney injury monitored by optical imaging. *International Journal of Molecular Medicine*.

[13] Shenoda B. B., Ajit S. K. (2016). Modulation of immune responses by exosomes derived from antigen-presenting cells. *Clinical Medicine Insights: Pathology*.

[14] Aswad H., Forterre A., Wiklander O. P. B. (2014). Exosomes participate in the alteration of muscle homeostasis during lipid-induced insulin resistance in mice. *Diabetologia*.

[15] Whiteside T. L. (2016). Exosomes and tumor-mediated immune suppression. *Journal of Clinical Investigation*.

[16] Johnsen K. B., Gudbergsson J. M., Skov M. N., Pilgaard L., Moos T., Duroux M. (2014). A comprehensive overview of exosomes as drug delivery vehicles—endogenous nanocarriers for targeted cancer therapy. *Biochimica et Biophysica Acta—Reviews on Cancer*.

[17] Raposo G., Nijman H. W., Stoorvogel W. (1996). B lymphocytes secrete antigen-presenting vesicles. *Journal of Experimental Medicine*.

[18] Sáenz-Cuesta M., Mittelbrunn M., Otaegui D. (2015). Editorial: novel clinical applications of extracellular vesicles. *Frontiers in Immunology*.

[19] Busato A., Bonafede R., Bontempi P. (2016). Magnetic resonance imaging of ultrasmall superparamagnetic iron oxide-labeled exosomes from stem cells: a new method to obtain labeled exosomes. *International Journal of Nanomedicine*.

[20] Smyth T., Kullberg M., Malik N., Smith-Jones P., Graner M. W., Anchordoquy T. J. (2015). Biodistribution and delivery efficiency of unmodified tumor-derived exosomes. *Journal of Controlled Release*.

[21] Hwang D. W., Choi H., Jang S. C. (2015). Noninvasive imaging of radiolabeled exosome-mimetic nanovesicle using 99m Tc-HMPAO. *Scientific Reports*.

[22] Lai C. P., Mardini O., Ericsson M. (2014). Dynamic biodistribution of extracellular vesicles in vivo using a multimodal imaging reporter. *ACS Nano*.

[23] Takahashi Y., Nishikawa M., Shinotsuka H. (2013). Visualization and in vivo tracking of the exosomes of murine melanoma B16-BL6 cells in mice after intravenous injection. *Journal of Biotechnology*.

[24] Fraker P. J., Speck J. C. (1978). Protein and cell membrane iodinations with a sparingly soluble chloroamide, 1,3,4,6-tetrachloro-3a,6a-diphenylglycoluril. *Biochemical and Biophysical Research Communications*.

[25] Pitt J. M., André F., Amigorena S. (2016). Dendritic cell-derived exosomes for cancer therapy. *Journal of Clinical Investigation*.

[26] Fais S. (2013). NK cell-released exosomes: natural nanobullets against tumors. *OncoImmunology*.

[27] Akao Y., Iio A., Itoh T. (2011). Microvesicle-mediated RNA molecule delivery system using monocytes/macrophages. *Molecular Therapy*.

[28] Goetzl E. J., Goetzl L., Karliner J. S., Tang N., Pulliam L. (2016). Human plasma platelet-derived exosomes: effects of aspirin. *FASEB Journal*.

[29] Danesh A., Inglis H. C., Jackman R. P. (2014). Exosomes from red blood cell units bind to monocytes and induce proinflammatory cytokines, boosting T-cell responses in vitro. *Blood*.

[30] Giusti I., Di Francesco M., Dolo V. (2016). Extracellular vesicles in glioblastoma: role in biological processes and in therapeutic applications. *Current Cancer Drug Targets*.

[31] Lee J. C., Zhao J.-T., Gundara J., Serpell J., Bach L. A., Sidhu S. (2015). Papillary thyroid cancer-derived exosomes contain miRNA-146b and miRNA-222. *Journal of Surgical Research*.

[32] Sandfeld-Paulsen B., Jakobsen K. R., Bæk R. (2016). Exosomal proteins as diagnostic biomarkers in lung cancer. *Journal of Thoracic Oncology*.

[33] Yang N., Li S., Li G. (2016). The role of extracellular vesicles in mediating progression, metastasis and potential treatment of hepatocellular carcinoma. *Oncotarget*.

[34] Zhang W., Yang J., Cao D., You Y., Shen K., Peng P. (2016). Regulation of exosomes released from normal ovarian epithelial cells and ovarian cancer cells. *Tumor Biology Journal*.

[35] Bigagli E., Luceri C., Guasti D., Cinci L. (2016). Exosomes secreted from human colon cancer cells influence the adhesion of neighboring metastatic cells: role of microRNA-210. *Cancer Biology & Therapy*.

[36] Deatherage B. L., Cookson B. T. (2012). Membrane vesicle release in bacteria, eukaryotes, and archaea: a conserved yet underappreciated aspect of microbial life. *Infection and Immunity*.

[37] An Q., Van Bel A. J. E., Hückelhoven R. (2007). Do plant cells secrete exosomes derived from multivesicular bodies?. *Plant Signaling and Behavior*.

[38] Imai T., Takahashi Y., Nishikawa M. (2015). Macrophage-dependent clearance of systemically administered B16BL6-derived exosomes from the blood circulation in mice. *Journal of Extracellular Vesicles*.

[39] Nordin J. Z., Lee Y., Vader P. (2015). Ultrafiltration with size-exclusion liquid chromatography for high yield isolation of extracellular vesicles preserving intact biophysical and functional properties. *Nanomedicine: Nanotechnology, Biology, and Medicine*.

[40] Ohno S.-I., Takanashi M., Sudo K. (2013). Systemically injected exosomes targeted to EGFR deliver antitumor microrna to breast cancer cells. *Molecular Therapy*.

[41] Varga Z., Gyurkó I., Pálóczi K. (2016). Radiolabeling of extracellular vesicles with 99mTc for quantitative in vivo imaging studies. *Cancer Biotherapy and Radiopharmaceuticals*.

[42] Morishita M., Takahashi Y., Nishikawa M. (2015). Quantitative analysis of tissue distribution of the B16BL6-derived exosomes using a streptavidin-lactadherin fusion protein and Iodine-125-Labeled biotin derivative after intravenous injection in mice. *Journal of Pharmaceutical Sciences*.

[43] Hu L., Wickline S. A., Hood J. L. (2015). Magnetic resonance imaging of melanoma exosomes in lymph nodes. *Magnetic Resonance in Medicine*.

[44] Urbanelli L., Magini A., Buratta S. (2013). Signaling pathways in exosomes biogenesis, secretion and fate. *Genes*.

[45] Klumperman J., Raposo G. (2014). The complex ultrastructure of the endolysosomal system. *Cold Spring Harbor Perspectives in Biology*.

[46] Beach A., Zhang H.-G., Ratajczak M. Z., Kakar S. S. (2014). Exosomes: an overview of biogenesis, composition and role in ovarian cancer. *Journal of Ovarian Research*.

[47] Théry C., Zitvogel L., Amigorena S. (2002). Exosomes: composition, biogenesis and function. *Nature Reviews Immunology*.

[48] Trajkovic K., Hsu C., Chiantia S. (2008). Ceramide triggers budding of exosome vesicles into multivesicular endosomes. *Science*.

[49] Henne W. M., Stenmark H., Emr S. D. (2013). Molecular mechanisms of the membrane sculpting ESCRT pathway. *Cold Spring Harbor Perspectives in Biology*.

[50] Ostrowski M., Carmo N. B., Krumeich S. (2010). Rab27a and Rab27b control different steps of the exosome secretion pathway. *Nature Cell Biology*.

[51] Cocucci E., Racchetti G., Meldolesi J. (2009). Shedding microvesicles: artefacts no more. *Trends in Cell Biology*.

[52] Vader P., Breakefield X. O., Wood M. J. A. (2014). Extracellular vesicles: emerging targets for cancer therapy. *Trends in Molecular Medicine*.

[53] El Andaloussi S., Mäger I., Breakefield X. O., Wood M. J. A. (2013). Extracellular vesicles: biology and emerging therapeutic opportunities. *Nature Reviews Drug Discovery*.

[54] Morse M. A., Garst J., Osada T. (2005). A phase I study of dexosome immunotherapy in patients with advanced non-small cell lung cancer. *Journal of Translational Medicine*.

[55] Escudier B., Dorval T., Chaput N. (2005). Vaccination of metastatic melanoma patients with autologous dendritic cell (DC) derived-exosomes: results of the first phase I clinical trial. *Journal of Translational Medicine*.

[56] Ophelders D. R. M. G., Wolfs T. G. A. M., Jellema R. K. (2016). Mesenchymal stromal cell-derived extracellular vesicles protect the fetal brain after hypoxia-ischemia. *Stem Cells Translational Medicine*.

[57] Nakano M., Nagaishi K., Konari N. (2016). Bone marrow-derived mesenchymal stem cells improve diabetes-induced cognitive impairment by exosome transfer into damaged neurons and astrocytes. *Scientific Reports*.

[58] Katakowski M., Buller B., Zheng X. (2013). Exosomes from marrow stromal cells expressing miR-146b inhibit glioma growth. *Cancer Letters*.

[59] Fonsato V., Collino F., Herrera M. B. (2012). Human liver stem cell-derived microvesicles inhibit hepatoma growth in SCID mice by delivering antitumor microRNAs. *Stem Cells*.

[60] Timmers L., Lim S. K., Hoefer I. E. (2011). Human mesenchymal stem cell-conditioned medium improves cardiac function following myocardial infarction. *Stem Cell Research*.

[61] Cho Y. B., Lee W. Y., Park K. J., Kim M., Yoo H.-W., Yu C. S. (2013). Autologous adipose tissue-derived stem cells for the treatment of crohn's fistula: A Phase I Clinical Study. *Cell Transplantation*.

[62] Garcia-Olmo D., Herreros D., Pascual I. (2009). Expanded adipose-derived stem cells for the treatment of complex perianal fistula: a phase II clinical trial. *Diseases of the Colon and Rectum*.

[63] Sun D., Zhuang X., Xiang X. (2010). A novel nanoparticle drug delivery system: the anti-inflammatory activity of curcumin is enhanced when encapsulated in exosomes. *Molecular Therapy*.

[64] Pardridge W. M. (2012). Drug transport across the blood-brain barrier. *Journal of Cerebral Blood Flow and Metabolism*.

[65] Harris D. A., Patel S. H., Gucek M., Hendrix A., Westbroek W., Taraska J. W. (2015). Exosomes released from breast cancer carcinomas stimulate cell movement. *PLoS ONE*.

[66] Yoon Y. J., Kim D.-K., Yoon C. M. (2014). Egr-1 activation by cancer-derived extracellular vesicles promotes endothelial cell migration via ERK1/2 and JNK signaling pathways. *PLoS ONE*.

[67] Simons M., Raposo G. (2009). Exosomes—vesicular carriers for intercellular communication. *Current Opinion in Cell Biology*.

[68] Skog J., Würdinger T., van Rijn S. (2008). Glioblastoma microvesicles transport RNA and proteins that promote tumour growth and provide diagnostic biomarkers. *Nature Cell Biology*.

[69] Koumangoye R. B., Sakwe A. M., Goodwin J. S., Patel T., Ochieng J. (2011). Detachment of breast tumor cells induces rapid secretion of exosomes which subsequently mediate cellular adhesion and spreading. *PLoS ONE*.

[70] Melo S. A., Luecke L. B., Kahlert C. (2015). Glypican-1 identifies cancer exosomes and detects early pancreatic cancer. *Nature*.

[71] Moon P.-G., Lee J.-E., Cho Y.-E. (2016). Identification of developmental endothelial locus-1 on circulating extracellular vesicles as a novel biomarker for early breast cancer detection. *Clinical Cancer Research*.

[72] Zomer A., Maynard C., Verweij F. J. (2015). In vivo imaging reveals extracellular vesicle-mediated phenocopying of metastatic behavior. *Cell*.

[73] Lassailly F., Griessinger E., Bonnet D. (2010). ‘Microenvironmental contaminations’ induced by fluorescent lipophilic dyes used for noninvasive in vitro and in vivo cell tracking. *Blood*.

[74] Li P., Zhang R., Sun H. (2013). PKH26 can transfer to host cells in vitro and vivo. *Stem Cells and Development*.

[75] Wiklander O. P. B., Nordin J. Z., O'Loughlin A. (2015). Extracellular vesicle in vivo biodistribution is determined by cell source, route of administration and targeting. *Journal of Extracellular Vesicles*.

[76] Parolini I., Federici C., Raggi C. (2009). Microenvironmental pH is a key factor for exosome traffic in tumor cells. *Journal of Biological Chemistry*.

[77] Ahn B.-C. (2014). Requisites for successful theranostics with radionuclide-based reporter gene imaging. *Journal of Drug Targeting*.

[78] Kim J. E., Kalimuthu S., Ahn B.-C. (2015). In vivo cell tracking with bioluminescence imaging. *Nuclear Medicine and Molecular Imaging*.

[79] Ottobrini L., Martelli C., Trabattoni D. L., Clerici M., Lucignani G. (2011). In vivo imaging of immune cell trafficking in cancer. *European Journal of Nuclear Medicine and Molecular Imaging*.

[80] Li X. J., Gangadaran P., Kalimuthu S. (2016). Role of pulmonary macrophages in initiation of lung metastasis in anaplastic thyroid cancer. *International Journal of Cancer*.

[81] Yoon J., Jo W., Jeong D., Kim J., Jeong H., Park J. (2015). Generation of nanovesicles with sliced cellular membrane fragments for exogenous material delivery. *Biomaterials*.

[82] Wahlgren J., Karlson T. D. L., Brisslert M. (2012). Plasma exosomes can deliver exogenous short interfering RNA to monocytes and lymphocytes. *Nucleic Acids Research*.

[83] Shtam T. A., Kovalev R. A., Varfolomeeva E. Y., Makarov E. M., Kil Y. V., Filatov M. V. (2013). Exosomes are natural carriers of exogenous siRNA to human cells *in vitro*. *Cell Communication and Signaling*.

[84] Hood J. L., San R. S., Wickline S. A. (2011). Exosomes released by melanoma cells prepare sentinel lymph nodes for tumor metastasis. *Cancer Research*.

[85] Hoffman R. M. (2013). Stromal-cell and cancer-cell exosomes leading the metastatic exodus for the promised niche. *Breast Cancer Research*.

[86] Suetsugu A., Honma K., Saji S., Moriwaki H., Ochiya T., Hoffman R. M. (2013). Imaging exosome transfer from breast cancer cells to stroma at metastatic sites in orthotopic nude-mouse models. *Advanced Drug Delivery Reviews*.

[87] Peinado H., Alečković M., Lavotshkin S. (2012). Melanoma exosomes educate bone marrow progenitor cells toward a pro-metastatic phenotype through MET. *Nature Medicine*.

[88] Mizrak A., Bolukbasi M. F., Ozdener G. B. (2013). Genetically engineered microvesicles carrying suicide mRNA/protein inhibit schwannoma tumor growth. *Molecular Therapy*.

[89] Chen L., Brigstock D. R. (2017). Cellular or exosomal microRNAs associated with CCN gene expression in liver fibrosis. *Methods in Molecular Biology*.

[90] Lai C. P.-K., Breakefield X. O. (2012). Role of exosomes/microvesicles in the nervous system and use in emerging therapies. *Frontiers in Physiology*.

[91] Choi H., Lee D. S. (2016). Illuminating the physiology of extracellular vesicles. *Stem Cell Research and Therapy*.

[92] Ahn B.-C. (2016). Personalized medicine based on theranostic radioiodine molecular imaging for differentiated thyroid cancer. *BioMed Research International*.

[93] Lee H. W., Yoon S. Y., Singh T. D. (2015). Tracking of dendritic cell migration into lymph nodes using molecular imaging with sodium iodide symporter and enhanced firefly luciferase genes. *Scientific Reports*.

[94] Lee H. W., Gangadaran P., Kalimuthu S., Ahn B.-C. (2016). Advances in molecular imaging strategies for *in vivo* tracking of immune cells. *BioMed Research International*.

[95] Lener T., Gimona M., Aigner L. (2015). Applying extracellular vesicles based therapeutics in clinical trials—an ISEV position paper. *Journal of Extracellular Vesicles*.

